# Association between Personal Activity Intelligence (PAI) and body weight in a population free from cardiovascular disease – The HUNT study

**DOI:** 10.1016/j.lanepe.2021.100091

**Published:** 2021-03-21

**Authors:** Sophie K. Kieffer, Javaid Nauman, Kari Syverud, Hege Selboskar, Stian Lydersen, Ulf Ekelund, Ulrik Wisløff

**Affiliations:** a Department of Circulation and Medical Imaging, Faculty of Medicine and Health Sciences, Norwegian University of Science and Technology, Postboks 8905, 7491 Trondheim, Norway; b Institute of Public Health, College of Medicine and Health Sciences, United Arab Emirates University, Al-Ain, United Arab Emirates; c Healthy Living for Pandemic Event Protection (HL-PIVOT) Network, Chicago, IL, USA; d Regional Centre for Child and Youth Mental Health and Child Welfare, Department of Mental Health, Faculty of Medicine and Health Sciences, Norwegian University of Science and Technology, Trondheim, Norway; e Department of Sports Medicine, Norwegian School of Sport Sciences, Oslo, Norway; f Department of Chronic Diseases and Ageing, Norwegian Institute of Public Health, Oslo, Norway; g School of Human Movement and Nutrition Science, University of Queensland, Queensland, Australia

## Abstract

**Background:**

Personal Activity Intelligence (PAI) is a new metric for physical activity tracking, and is associated with reduced risk of all-cause and cardiovascular mortality. We prospectively investigated whether PAI is associated with lower body weight gain in a healthy population.

**Methods:**

We included 85,243 participants (40,037 men and 45,206 women) who participated in at least one of three waves of the Trøndelag Health Study (HUNT1: 1984-86, HUNT2: 1995-97, and HUNT3: 2006-08). We used questionnaires to estimate PAI, and linear mixed models to examine body weight according to PAI levels at three study waves. We also conducted regression analyses to assess separate relationships between change in PAI and the combined changes in PAI and physical activity recommendations, according to body weight from HUNT1 to HUNT3.

**Findings:**

Compared with HUNT1, body weight was 8.6 and 6.7 kg higher at HUNT3 for men and women, respectively, but was lower among those with ≥200 PAI at HUNT3. For both sexes, a change from inactive (0 PAI) at HUNT1 to ≥100 weekly PAI-score at HUNT2 and HUNT3, and a ≥100 PAI-score at all three occasions were associated with lower body weight gain, compared with the reference group (0 PAI at all three waves). Importantly, among both sexes, obtaining ≥100 weekly PAI at HUNT1 and HUNT3 was associated with lower body weight gain regardless of adhering to physical activity guidelines.

**Interpretation:**

Adhering to a high PAI over time may be a useful tool to attenuate excessive body weight gain in a population free from cardiovascular disease.

**Funding:**

Norwegian Research Council and the Liaison Committee between the Central Norway Regional Health Authority and the Norwegian University of Science and technology.

**Research in context**

**Evidence before this study**

We searched PubMed for clinical and observational studies, meta-analyses, and review articles published until December 2020, using the following search terms: “physical activity”, “exercise”, “change in physical activity/exercise”, “body weight”, “weight gain”, “weight gain prevention”, “exercise/physical activity”, and “body weight/weight gain prevention”. The scientific evidence supports that regular exercise or physical activity is an effective strategy to minimize or prevent weight gain in adults.

**Added value of this study**

Our study reports an association between Personal Activity Intelligence (PAI) and weight gain over time. PAI is a novel activity metric which is based on mechanistic interactions between physical activity and fitness using relative intensity physical activity. Although, body weight increased over time in both men and women regardless of PAI, the weight gain was least pronounced in participants who maintained or increased their PAI scores over time. Interestingly, when PAI scores were taken into account, there were no significant differences in body weight regardless of following the physical activity recommendations. Furthermore, our findings show the PAI metric's importance when used in health risk assessments. Activity data can be shared between clinicians and patients/consumers and provide an opportunity for the clinicians to both track the activity levels of their patients and motivate them to increase their activity levels to improve health outcomes.

**Implications of all the available evidence**

Over time, individuals gain body weight regardless of physical activity engagement. However, available data show that body weight is modifiable, and support the fact that moderate to high intensity exercise may be a cost-effect strategy to minimise the impact of the obesity pandemic. Our results indicate that individuals may be able to prevent or minimise weight gain by obtaining high PAI scores during a week. The PAI metric may be an appropriate and personalised metric for both healthy people and individuals with excessive body weight to motivate physical activity participation and mitigate weight gain.

Alt-text: Unlabelled box

## 1 Introduction

The number of obese individuals has tripled over the last 50 years, reaching epidemic proportions [1,2]. Data suggest that excessive body weight has contributed to 4·7 million deaths worldwide in 2017, [3] representing a major public health concern. Excessive body weight results from an imbalance between energy intake and expenditure [4]. Therefore, adults are encouraged to limit energy intake, and engage in regular physical activity [1,5]. The current physical activity guidelines for adults consist of 150 to 300 weekly minutes of moderate intensity physical activity, or 75 to 150 weekly minutes of vigorous intensity physical activity, or a combination of both [5,6,7]. Even though meeting these guidelines has been related to lower body weight and improved health outcomes [[8], [9], [10], [11], [12]], adherence remains low [6,[13], [14], [15]]. Indeed, the guidelines define intensity both in relative terms (relative to one's cardiorespiratory capacity) such as percentage of heart rate reserve (%HRR, exercise heart rate above resting heart rate relative to the difference between resting and maximal heart rate), and absolute terms such as km/h or METs (metabolic equivalent of tasks) [6,16,17]. For instance, walking at 5 km/h equates moderate intensity in absolute terms which may lead to confusion as it equates vigorous intensity in relative terms when performed by an individual with low cardiorespiratory fitness (CRF) [18]. It may further be problematic as a 5 km/h pace may be above an individual's maximal CRF [18]. Considering that individuals with excessive body weight tend to have low CRF relative to their body mass,[19] relative exercise intensity may be more appropriate when quantifying and advocating physical activity goals for these individuals.

Recently, an activity metric named Personal Activity Intelligence (PAI) was developed based on mechanistic interactions between physical activity and CRF [20,21]. The metric accounts for relative intensity of physical activity through %HRRs: when combined with a heart rate monitor, the metric translates individual weekly heart rates, by the means of individual resting and maximal heart rates, into a straightforward and easily applicable sex-specific and personal score [[20], [21], [22], [23], [24]]. Thus, PAI scores may be obtained while performing different combinations of physical activity options at varying intensities according to personal preferences, as long as individual heart rates are elevated above resting heart rate. For example, a score of 100 PAI can be obtained by combining 60 weekly minutes of brisk walking, 40 weekly minutes of cycling, 50 weekly minutes of swimming, 30 weekly minutes of dancing/aerobics, and 20 weekly minutes of running [24]. A weekly PAI-score of 0 represents inactivity, whereas a weekly ≥100 PAI-score was associated with lower risk of all-cause and cardiovascular disease (CVD) mortality among adults in the general population, in adults with established CVD, and in sub-group analysis of adults with overweight and obesity [[20], [21], [22], [23], [24], [25]].

However, the long-term association between the activity metric of PAI and body weight is not known. A relationship between PAI and prevention of weight gain over the years may contribute to the knowledge regarding the general protective effect of PAI on mortality. Therefore, we aimed to evaluate the association between PAI and body weight at three occasions, and to test the hypothesis that change in PAI over time is associated with change in body weight.

## 2 Methods

### 2.1 Study population

**Fig. 1:**
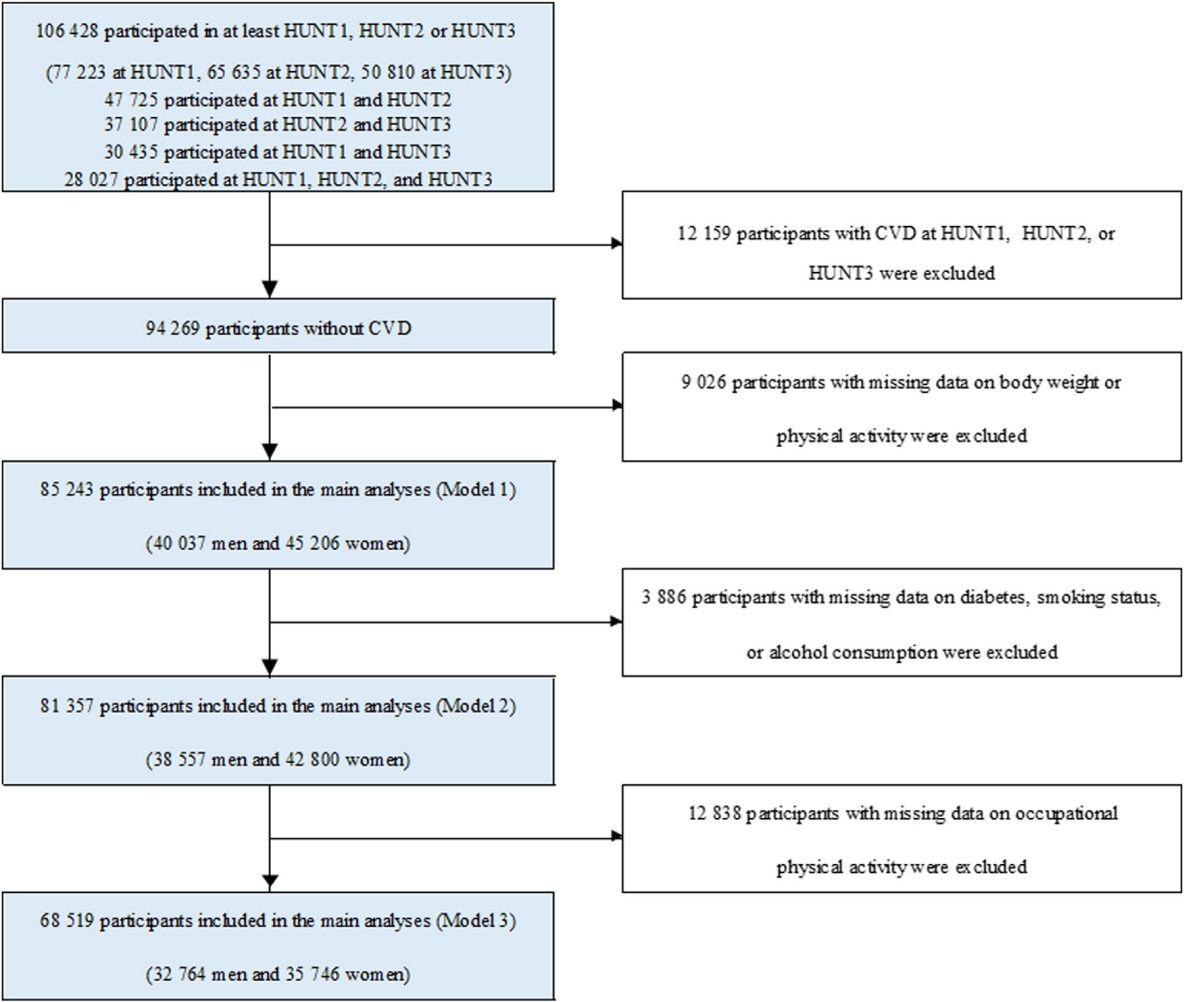
Flow of participants in the study CVD: cardiovascular disease, kg; kilogram, HUNT; The Trøndelag Health Study.

We used data from a Norwegian population study, the Trøndelag Health Study (the HUNT Study) in which participants are followed up longitudinally between the surveys, and in several virtually complete national health registries. The detailed account of the HUNT Study has been described elsewhere [26]. Briefly, the entire population aged 20 years and older from the Trøndelag County in Norway was invited to participate in three waves of the HUNT study: HUNT1 (1984-1986), HUNT2 (1995-1997), and HUNT3 (2006-2008). A total of 77 223 (89·4%) individuals at HUNT1, 65 635 (69·5%) at HUNT2 and 50 810 (54·1%) at HUNT3 accepted the invitations. For the present study, we selected 106 428 unique individuals who participated in at least one of three HUNT waves. Among these individuals with more than 193 000 observations across all study waves, 47 725 participated in both HUNT1 and HUNT2, 37 107 participated in both HUNT2 and HUNT3, 30 435 participated in both HUNT1 and HUNT3, and 28 027 participated in all three HUNT waves. We wanted to study the association between PAI and body weight in relatively healthy participants, therefore, 12 159 participants with self-reported CVD (angina pectoris, myocardial infarction, or stroke) at any time point of three HUNT waves were excluded. Further, 7 039 participants with missing information on physical activity, 1 987 participants with missing information on body weight, 3 886 participants with missing data on diabetes, smoking status, or alcohol consumption, and 12 838 participants with missing data on occupational physical activity were excluded when these data were missing at all three HUNT waves. This resulted in 68 519 participants in our main analyses (Model 3, Fig. 1). All participants provided informed written consents. The study was approved by the regional Committee for Medical and Health Ethics (2017/319/REKmidt).

### 2.2 Clinical and questionnaire-based information

Participants filled out questionnaires and went through a clinical examination at all three HUNT waves[26]. In the analyses, we used information from the questionnaires to obtain data regarding participants’ physical activity levels, age, smoking status, diabetes status, use of antihypertensive drugs and alcohol consumption. Trained nurses measured brachial artery blood pressure, height and body weight using standardized methods [26]. The body weight was measured to the nearest half kilogram without shoes while wearing light clothing without jackets, and outdoor garments [26,27].

### 2.3 Personal Activity Intelligence (PAI)

Information on leisure time physical activity was obtained from a self-administered questionnaire. PAI scores for each participant at all three HUNT waves were estimated using the responses to physical activity questions about duration, frequency and relative intensity [[20], [21], [22], [23],^25^]. The major assumptions underlying the PAI metric have robust scientific background and include a threshold of exercise intensity after which PAI can be accumulated, as very low intensity does not contribute to increased cardiorespiratory fitness [20]. The metric further includes a non-linear scaling of exercise intensity, as fewer exercise sessions of higher intensities are associated with similar or improved health benefits compared with frequent sessions at low intensity activity [20]. Finally, it is easier to earn the first 50 PAI vs the next 50 PAI because of an exercise induced lowering of resting as well as submaximal or maximal heart rates, and the evidence that moving from an inactive state to an active one is associated with a relatively larger reduction in mortality compared with moving from a relatively active to a very active state [20].

For each participant at HUNT1 and HUNT3, average weekly physical activity frequency and duration were multiplied to obtain weekly minutes spent performing physical activity [20]. Frequency of physical activity was assessed with “How often do you exercise? (on the average)”. The different options: “Never”, “Less than once a week”, “Once a week, 2-3 times a week”, “Nearly every day” were translated to 0, 0.5, 1, 2.5, and 5 weekly days, respectively. Physical activity duration was assessed with “For how long do you exercise each time?”. The following options: “Less than 15 minutes”, “16-30 minutes”, “30 minutes-1 hour”, “More than 1 hour” were translated to 7.5, 22.5, 45, and 60 minutes. Intensity of exercise which was stated as "How hard do you exercise?" provided three options ("no sweat or heavy breath," "heavy breath and sweat," and "push myself to exhaustion") [28]. The physical activity questions at HUNT2 distinguished between light (“no sweat/not out of breath”) and hard (“sweating/out of breath”) physical activity during a week, providing four response options for each intensity level (none, less than one hour, one to two hours or at least three hours) [29]. To estimate the weekly PAI score, we combined weekly minutes with physical activity intensities using %HRRs [20]. According to the PAI algorithm, and based on previous studies, these exercise intensities of “no sweat”, “sweating/heavy breathing” and “practically exhaustion” correspond to approximately 44%, 73% and 83% of HRR, respectively [20,21]. Because the questions about light and hard physical activity at HUNT2 were not mutually exclusive, the number of inactive participants at HUNT 2 were lower compared with the other two HUNT waves (eTable 1).

The physical activity questions were further used to estimate MET-hours per week where weekly minutes spent performing physical activity were divided by 60 to obtain weekly hours, and further multiplied with METs. Similar to a previous study, [30] the three intensity options were respectively translated to 3, 6, and 9 METs.

### 2.4 Statistical analyses

Descriptive data are reported as mean (standard deviation) for continuous variables and number (%) for categorical variables. To examine the association between PAI and body weight, participants were divided into five groups according to their weekly PAI-scores: 0 PAI (inactive), 1 to 50 PAI, 51 to 99 PAI, 100 to 199 PAI and ≥200 PAI, using the inactive group as reference category. The choice of these cut points was made “a priori” based on previous reports, [[20], [21], [22], [23], [24], [25]] and further extended to investigate the association between PAI and body weight. Linear mixed model analyses [31] were performed to examine body weight according to PAI levels at all three HUNT waves using data from participants who underwent at least one of the three HUNT waves. The estimates of linear mixed models are unbiased under the missing at random (MAR) assumption while a complete case analysis would have been unbiased only under the more restrictive missing completely at random (MCAR) assumption. Previous studies have suggested that HUNT data are not MCAR [26,32]. The dependent variable (weight in kg) and all covariates were updated at all three HUNT waves when available. Three models were used based on a previous study assessing the relationship between physical activity and body weight in the HUNT population^9^: model 1 included PAI, period indicator (HUNT1, HUNT2 and HUNT3), an interaction term between PAI and period indicator, and age (continuous variable); model 2 further included diabetes status (yes or no), smoking status (never, former or current), and alcohol consumption (abstainers, <1 time/2 weeks, 1-4 times/2 weeks or ≥5 times/2 weeks), and model 3 further included occupational physical activity (sedentary, walk and lift, and heavy). For our analyses, the number of participants is different across the three statistical models depending upon the variables used in these models (Fig. 1).

We also conducted age adjusted linear regression analyses for participants who underwent all three HUNT waves, with complete data on weight, PAI and age. We used difference in body weight from HUNT1 to HUNT3 as dependent variable [31]. For the analysis, we divided PAI into 3 categories at each of the three HUNT waves (0 PAI, 1-99 PAI and ≥100 PAI), constructing 27 different PAI change categories.

In a separate analysis, participants were divided according to whether or not they obtained ≥100 weekly PAI and whether or not they performed ≥7·5 MET-hours corresponding to meeting the lower level of the current physical activity recommendations (i.e. 150 min of moderate or 75 minutes of vigorous intensity), [5,6,11] at both HUNT1 and HUNT3. This resulted in 16 different categories.

Finally, we conducted sensitivity analyses where only individuals with complete information on all covariates who participated in all three HUNT waves and were free from CVD were included, i.e., complete case analysis (eFig. 1). We report 95% confidence intervals (CI) where relevant, and two-sided *P*-values <0.05 as indications of statistical significance. We used Stata statistical software (version 15.1, StataCorp, College Station, TX, USA) for all statistical analyses.

Role of the funding source: The funding organizations had no role in the design and conduct of the study, in the collection, analysis, and interpretation of the data or in the preparation, review, or approval of the manuscript.

## 3 Results

Baseline characteristics of men and women according to PAI levels are presented in Table 1. For both sexes, participants with high PAI-scores were younger and had a healthier risk profile compared with their inactive counterparts, i.e., lower body weight, lower percentage of participants with high alcohol consumption and hypertension (systolic blood pressure above 139 mmHg, diastolic blood pressure above 89 mmHg or use of antihypertensive drugs), fewer number of current smokers, and lower percentage of participants with diabetes. Over the 22 years of follow-up of those individuals with data on body weight at HUNT1 and HUNT 3, we observed an overall increase in body weight of 8·0 kg (95% CI: 7·8 to 8·1) and 8·5 kg (95% CI: 8·3 to 8·6) for men and women, respectively, with the largest increase observed during the first 11 years (4·6 kg, 95% CI: 4·5 to 4·7 for men, 5·1 kg, 95% CI: 5·0 to 5·2 for women**)** for those with data on body weight at HUNT1 and HUNT2. A detailed description of the individual's characteristics, pooling men and women, according to participation in three HUNT waves is presented in eTable 1.

**Table 1:** Characteristics of study participants according to baseline (HUNT1) PAI levels (N=51 004)^a^.

	Men					Women			
	Inactive	PAI ≤50	PAI 51-99	PAI 100-199	PAI ≥200	PAI ≤50	PAI 51-99	PAI 100-199	PAI ≥200
	(N= 10 381)	(N= 6007)	(N= 2 104)	(N= 3 229)	(N= 2 605)	(N= 9 519)	(N=2 744)	(N= 968)	(N= 2 346)
Age years, mean (SD)	45.8 (17.1)	53.8 (17.3)	46.0 (16.6)	42.1 (13.7)	39.7 (16.3)	50.7 (17.0)	44.6 (15.9)	38.1 (12.4)	38.8 (13.1)
Weight kg, mean (SD)	78.8 (11.7)	78.0 (11.0)	78.4 (10.2)	78.4 (9.7)	77.5 (9.3)	66.5 (11.5)	64.7 (10.5)	64.2 (9.9)	64.9 (9.9)
Height cm, mean (SD)	176.6 (6.7)	175.5 (6.9)	177.0 (6.7)	177.9 (6.5)	177.9 (6.6)	162.8 (6.3)	164.1 (6.1)	164.8 (5.7)	165.1 (5.9)
BMI, kg/m^2^, N (%)									
< 18.5	87 (0.8)	32 (0.5)	10 (0.5)	5 (0.2)	3 (0.1)	171 (1.8)	46 (1.7)	18 (1.9)	38 (1.6)
18.5 - 24.9	5 102 (49.2)	2 891 (48.1)	1 099 (52.2)	1 866 (57.8)	1 610 (61.8)	5 124 (53.8)	1 816 (66.2)	688 (71.1)	1 617 (68.9)
25.0 - 29.9	4 187 (40.3)	2591 (43.1)	885 (42.1)	1 216 (37.7)	909 (34.9)	2 957 (31.1)	660 (24.1)	194 (20.0)	546 (23.3)
≥ 30.0	919 (8.9)	480 (8.0)	110 (5.2)	137 (4.2)	83 (3.2)	1 234 (13.0)	218 (7.9)	66 (6.8)	142 (6.1)
Alcohol consumption/ 2 weeks, N (%)									
Abstainer	615 (5.9)	492 (8.2)	118 (5.6)	147 (4.6)	140 (5.4)	1 500 (15.8)	298 (10.9)	69 (7.1)	169 (7.2)
<1 time	3 674 (35.4)	2 407 (40.1)	691 (32.8)	994 (30.8)	798 (30.6)	4 908 (51.6)	1 298 (47.3)	449 (46.4)	1 067 (45.5)
1-4 times	4 941 (47.6)	2 489 (41.4)	1 067 (50.7)	1 778 (55.1)	1 450 (55.7)	2 489 (26.2)	990 (36.1)	414 (42.8)	979 (41.7)
≥ 5 times	965 (9.3)	458 (7.6)	184 (8.8)	282 (8.7)	181 (7.0)	254 (2.7)	92 (3.4)	25 (2.6)	104 (4.4)
Smoking status, N (%)									
Never	2 762 (26.6)	1 788 (29.8)	799 (38.0)	1 403 (43.5)	1 331 (51.1)	4 827 (50.7)	1 309 (47.7)	459 (47.4)	1 154 (49.2)
Former	2 657 (25.6)	1 866 (31.1)	631 (30.0)	1 025 (31.7)	639 (24.5)	1 492 (15.7)	530 (19.3)	190 (19.6)	545 (23.2)
Current	4 633 (44.6)	2 131 (35.5)	634 (30.1)	742 (23.0)	576 (22.1)	2 676 (28.1)	808 (29.5)	304 (31.4)	608 (25.9)
Hypertension status, N (%)									
Yes	5 150 (49.6)	3 557 (59.2)	1 073 (51.0)	1 401 (43.4)	1 077 (41.3)	4 506 (47.3)	923 (33.6)	208 (21.5)	586 (25.0)
No	5 192 (50.0)	2 439 (40.6)	1 026 (48.8)	1 815 (56.2)	1 522 (58.4)	4 997 (52.5)	1 819 (66.3)	759 (78.4)	1 758 (74.9)
Diabetes status, N (%)									
Yes	200 (1.9)	156 (2.6)	43 (2.0)	31 (1.0)	33 (1.3)	234 (2.5)	36 (1.3)	8 (0.8)	15 (0.6)
No	10 168 (98.0)	5 850 (97.4)	2 061 (98.0)	3 195 (99.0)	2 572 (98.7)	9 283 (97.5)	2 707 (98.7)	960 (99.2)	2 331 (99.4)
Occupational PA, N (%)									
Sedentary	1001 (9.6)	572 (9.5)	300 (14.3)	604 (18.7)	376 (14.4)	806 (8.5)	284 (10.4)	99 (10.2)	312 (13.3)
Walk and lift	5 946 (57.3)	2 820 (47.0)	1 204 (57.2)	1 972 (61.1)	1 459 (56.0)	5 065 (53.2)	1 637 (59.7)	623 (64.4)	1 457 (62.1)
Heavy	983 (9.5)	284 (4.7)	107 (5.1)	177 (5.5)	197 (7.6)	536 (5.6)	154 (5.6)	68 (7.0)	154 (6.6)

PAI: Personal Activity Intelligence, kg: kilograms, m: meters.

a 14 888 participants at HUNT1 had missing data on PAI.

### 3.1 PAI and changes in body weight using linear mixed model analysis

**Fig. 2:**
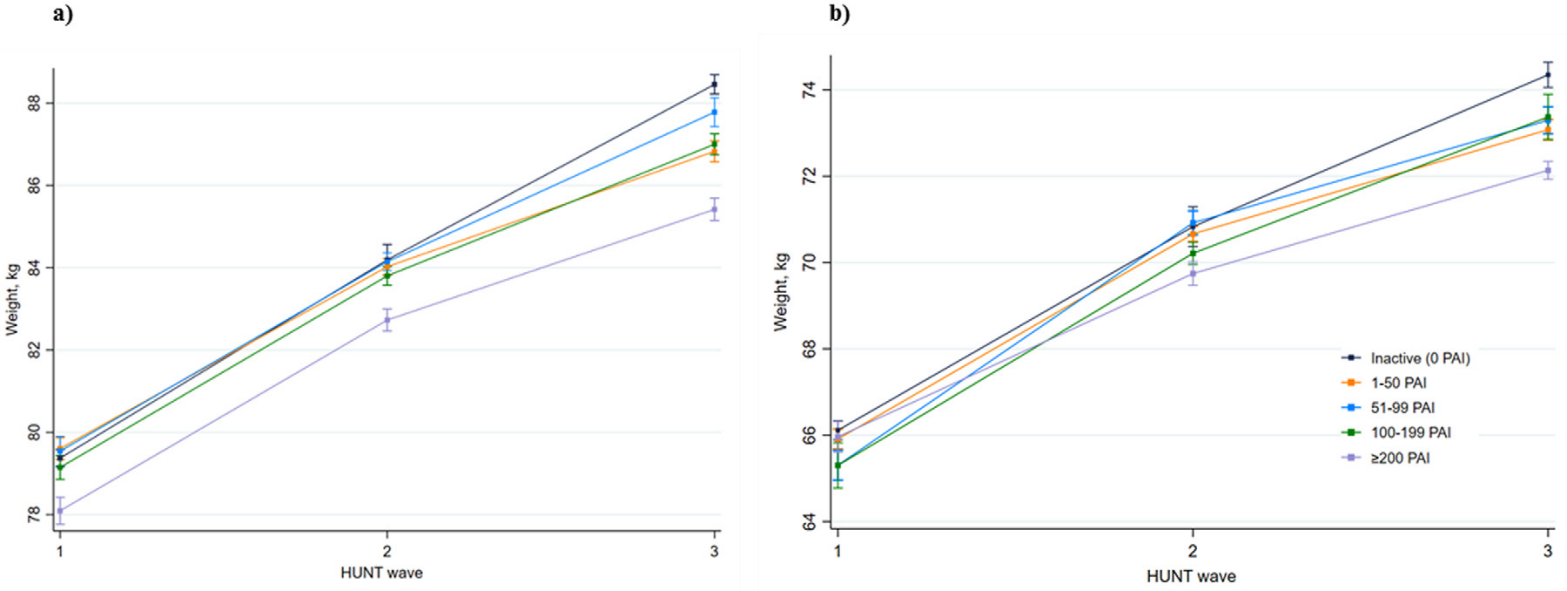
Body weight according to PAI categories across HUNT waves. a) Men; b) Women PAI; Personal Activity Intelligence, kg; kilogram, HUNT; The Trøndelag Health Study. The estimates of body weight for participants who maintained the same levels of PAI across HUNT waves: adjusted for age, diabetes (yes or no), smoking status (never, former, current), alcohol consumption (abstainers, <1 time/2 weeks, 1-4 times/2weeks or ≥5 times/2weeks) and hypertension (yes or no) and occupational physical activity (sedentary, walk and lift and heavy). The squares represent mean weight in kilograms, and error bars represents 95% confidence intervals.

Among both sexes, body weight at HUNT3 was higher across all PAI groups compared with the body weight at HUNT1, and weight gain over time was most pronounced among inactive individuals at HUNT1 (eTable 2 & Table 2). Based on the results from linear mixed model analyses including data of individuals who participated in at least one of the three HUNT waves, body weight was on average 8.6 kg (95% CI: 8.4 to 8.9) and 6.7 kg (95% CI: 6.5 to 6.9) higher at HUNT3 compared with HUNT1, for men and women respectively (data not shown, unadjusted analyses). Compared with the reference group of inactive men at HUNT1, body weight was 4·8 kg (95% CI: 4·4 to 5·2) higher at HUNT2 among inactive men and 9·1 kg (95% CI: 8·8 to 9·4) higher at HUNT3 among inactive men (Table 2, Fig. 2a). However, body weight was 1·3 kg (95% CI: 0·9 to 1·6) lower at HUNT1 among men with a weekly PAI-score of ≥200, compared with inactive men at HUNT1 (Table 2, Fig. 2a). Among men with a weekly PAI-score of ≥200 at HUNT3, the increase in body weight was 1·8 kg (95% CI: 1·3 to 2·2) lower than the increase in body weight of inactive men at the same time point (Table 2, Fig. 2a).

**Table 2:** Difference in body weight (95% CI) in kilogram and interaction estimates between body weight and time by PAI categories.

		Men				Women			
	PAI	N	HUNT1	HUNT2	HUNT3	N	HUNT1	HUNT2	HUNT3
**Model 1**	Inactive		Ref	5.0 (4.6 to 5.3)	10.0 (9.7 to 10.2)		Ref	4.2 (3.8 to 4.5)	7.6 (7.3 to 7.9)
	≤50		0.5 (0.3 to 0.8)	*-0.6 (-1.0 to -0.2)*	*-2.4 (-2.7 to -2.0)*		0.4 (0.2 to 0.6)	*-0.1 (-0.5 to 0.3)*	*-1.5 (-1.9 to -1.2)*
	51-99		0.1 (-0.2 to 0.5)	*0.2 (-0.3 to 0.7)*	*-0.6 (-1.1 to -0.1)*		-0.6 (-1.0 to -0.3)	*1.4 (0.9 to 1.9)*	*-0.2 (-0.6 to 0.3)*
	100-199		0.1 (-0.2 to 0.3)	*0.7 (-0.4 to 0.5)*	*-1.0 (-1.4 to -0.6)*		-0.8 (-1.3 to -0.3)	*0.8 (0.2 to 1.4)*	*1.0 (0.3 to 1.7)*
	≥200		-1.1 (-1.4 to -0.8)	*0.0 (-0.5 to 0.5)*	*-1.6 (-2.0 to -1.2)*		-0.3 (-0.6 to 0.0)	*-0.2 (-0.7 to 0.3)*	*-0.7 (-1.1 to -0.3)*
		40 037				45 206			
**Model 2**	Inactive		Ref	4.9 (4.5 to 5.2)	9.5 (9.2 to 9.7)		Ref	4.1 (3.7 to 4.4)	7.6 (7.3 to 7.9)
	≤50		0.4 (0.2 to 0.7)	*-0.5 (-1.0 to -0.1)*	*-2.4 (-2.7 to -2.0)*		0.3 (0.0 to 0.5)	*0.1 (-0.3 to 0.6)*	*-1.6 (-1.9 to -1.2)*
	51-99		0.0 (-0.3 to 0.4)	*0.2 (-0.3 to 0.7)*	*-0.7 (-1.2 to -0.2)*		-0.8 (-1.1 to -0.4)	*1.5 (1.0 to 2.1)*	*-0.3 (-0.7 to 0.2)*
	100-199		-0.2 (-0.5 to 0.1)	*0.1 (-0.3 to 0.6)*	*-0.9 (-1.3 to -0.6)*		-0.9 (-1.4 to -0.4)	*0.1 (0.3 to 1.6)*	*0.8 (0.0 to 1.5)*
	≥200		-1.3 (-1.7 to -1.0)	*0.0 (-0.4 to 0.5)*	*-1.6 (-2.0 to -1.2)*		-0.5 (-0.9 to -0.2)	*0.0 (-0.5 to 0.5)*	*-0.9 (-1.3 to -0.5)*
		38 557				42 800			
**Model 3**	Inactive		Ref	4.8 (4.4 to 5.2)	9.1 (8.8 to 9.4)^a^		Ref	4.7 (4.2 to 5.2)	8.2 (7.9 to 8.6)
	≤50		0.2 (0.0 to 0.5)	*-0.4 (-0.9 to 0.1)*	*-1.9 (-2.3 to -1.5)*		-0.2 (-0.5 to 0.1)	*0.0 (-0.5 to 0.6)*	*-1.1 (-1.5 to -0.7)*
	51-99		0.2 (-0.2 to 0.5)	*-0.2 (-0.7 to 0.3)*	*-0.8 (-1.4 to -0.3)*		-0.8 (-1.2 to -0.4)	*0.9 (0.3 to 1.5)*	*-0.2 (-0.8 to 0.3)*
	100-199		-0.2 (-0.5 to 0.1)	*-0.2 (-0.6 to 0.3)*	*-1.2 (-1.6 to -0.8)*		-0.8 (-1.3 to -0.3)	*0.2 (-0.5 to 0.9)*	*-0.2 (-0.9 to 0.6)*
	≥200		-1.3 (-1.6 to -0.9)^a^	*-0.2 (-0.7 to 0.3)*	*-1.8 (-2.2 to -1.3)^a^*		-0.2 (-0.5 to 0.2)	*-0.9 (-1.5 to -0.3)*	*-2.1 (-2.5 to -1.6)*
		32 764				35 746			

PAI: Personal Activity Intelligence, HUNT: The Trøndelag Health Study, CI: confidence interval, N: number

Model 1 adjusted for age.

Model 2 was further adjusted for diabetes status (yes or no), smoking status (never, former, current) and alcohol consumption (abstainers, <1 time/2 weeks, 1-4 times/2weeks or ≥5 times/2weeks).

Model 3 was further adjusted for occupational physical activity (sedentary, walk and lift and heavy).

Numbers in italic are interactions estimates between PAI category and HUNT wave. The interpretation is as follows: ^a^Compared with the inactive group at HUNT1, those obtaining ≥200 at HUNT1 has 1.3 kg lower body weight at HUNT1. Compared to the same reference group, inactive men had 9.1 kg higher body weight at HUNT3, and men obtaining ≥200 at HUNT3 had 1.8 kg lower body weight increase at HUNT3 (interaction estimate).

Finally, the expected body weight in the ≥200 PAI group (eTable 2) can then be calculated with the following equation: body weight in reference group – 1.3 + 9.1 – 1.8.

Among inactive women, body weight was 4·7 kg (95% CI: 4·2 to 5·2) higher at HUNT2 and 8·2 kg (95% CI: 7·9 to 8·6) higher at HUNT3, compared with the reference group of inactive women at HUNT1 (Table 2, Fig. 2b). Among women with weekly PAI-score of ≥200 at HUNT1, body weight was 0·2 kg (95% CI: −0·2 to 0·5) lower (Table 2, Fig. 2b), compared with the same reference group. Among women with a weekly PAI-score of ≥200 at HUNT3, the increase in body weight was 2·1 kg (95% CI: 1·6 to 2·5) lower than the increase in body weight of inactive women at HUNT3 (Table 2, Fig. 2b). The difference in body weight in absolute kilograms related to different levels of PAI-score is presented in eTable 3. Compared with men obtaining ≥200 PAI at HUNT1, body weight was 7·3 kg (95% CI: 6·9 to 7·7) higher at HUNT3 among men with a weekly PAI-score of ≥200 at HUNT3, and the corresponding estimate for women was 6·2 kg (95% CI: 5·8 to 6·6), (eTable 3). Furthermore, results from the complete case analysis for both men and women were not materially different than our main results, except from showing a larger inverse association between PAI and body weight at HUNT2 (eTable 4).

**Table 3:** Change in body weight according to change in Personal Activity Intelligence.

Personal Activity Intelligence			Men		Women	
HUNT1	HUNT2	HUNT3	N	Weight change (CI)	N	Weight change (CI)
0	0	0	228	Ref	166	Ref
0	0	1-99	100	0.3 (-1.5 to 2.0)	130	-0.8 (-2.7 to 1.1)
0	0	≥100	39	0 (-2.5 to 2.5)	41	-0.9 (-3.7 to 2.0)
0	1-99	0	891	0.5 (-0.6 to 1.5)	674	0 (-1.5 to 1.4)
0	1-99	1-99	938	-0.4 (-1.5 to 0.7)	1 437	-1.1 (-2.5 to 0.2)
0	1-99	≥100	476	-0.3 (-1.5 to 0.9)	676	-1.5 (-3.0 to -0.1)
0	≥100	0	231	0.5 (-0.9 to 1.8))	129	-0.1 (-2.1 to 1.8)
0	≥100	1-99	252	-0.3 (-1.7 to 1.0)	269	-1.4 (-3.0 to 0.2)
0	≥100	≥100	269	-1.2 (-2.5 to 0.2)	333	-2.3 (-3.8 to -0.7)
1-99	0	0	28	0.1 (-2.9 to 3.0)	51	-0.8 (-3.4 to 1.9)
1-99	0	1-99	32	-0.5 (-3.3 to 2.3)	59	-2.1 (-4.5 to 0.4)
1-99	0	≥100	6	-4.3 (-10.4 to 1.7)	27	-1.2 (-4.6 to 2.2)
1-99	1-99	0	291	0.2 (-1.1 to 1.5)	395	-0.4 (-2.0 to 1.1)
1-99	1-99	1-99	810	-0.1 (-1.2 to 1.0)	1 946	-1.4 (-2.7 to 0)
1-99	1-99	≥100	448	-0.6 (-1.8 to 0.6)	879	-2.0 (-3.4 to -0.6)
1-99	≥100	0	84	-0.7 (-2.5 to 1.2)	70	0.1 (-1.3 to 3.3)
1-99	≥100	1-99	268	-0.1 (-1.4 to 1.3)	555	-1.4 (-2.8 to 0.1)
1-99	≥100	≥100	386	-1.7 (-2.9 to -0.4)	660	-2.6 (-4.0 to -1.1)
≥100	0	0	18	-0.1 (-3.7 to 3.5)	7	-2.0 (-8.3 to 4.3)
≥100	0	1-99	16	0.6 (-3.2 to 4.4)	13	2.1 (-2.6 to 6.9)
≥100	0	≥100	25	0.7 (-2.4 to 3.7)	4	-0.1 (-8.4 to 8.2)
≥100	1-99	0	169	2.0 (0.6 to 3.5)	83	-0.7 (-2.9 to 1.5)
≥100	1-99	1-99	371	0.3 (-0.9 to 1.6)	383	-1.3 (-2.8 to 0.3)
≥100	1-99	≥100	478	-1.0 (-2.2 to 0.2)	420	-2.3 (-3.8 to -0.8)
≥100	≥100	0	94	1.5 (-0.3 to 3.3)	31	2.9 (-0.3 to 6.1)
≥100	≥100	1-99	313	-0.6 (-1.8 to 0.7)	214	-2.3 (-4.0 to -0.6)
≥100	≥100	≥100	1091	-1.6 (-2.6 to -0.5)	627	-3.9 (-5.4 to -2.5)

HUNT: The Trøndelag Health Study, CI: confidence interval.

### 3.2 Changes in PAI, physical activity recommendations and body weight

For both men and women, those remaining active with ≥100 weekly PAI and those increasing their activity from inactive to ≥100 weekly PAI had lower body weight gain, compared with those remaining inactive at all three HUNT waves. Men who increased their activity level from inactive at HUNT1 to ≥100 weekly PAI at HUNT2 and HUNT3 had 1·2 kg (95% CI: −0·2 to 2·5) less body weight gain at HUNT3 (Table 3), whereas men with ≥100 weekly PAI at all three HUNT waves had 1·6 kg (95% CI: 0·5 to 2·6) lower body weight gain at HUNT3 (Table 3). Among women, those who increased their activity level from inactive at HUNT1 to ≥100 weekly PAI at HUNT2 and HUNT3 had 2·3 kg (95% CI: 0·7 to 3·8) less body weight gain at HUNT3, whereas women with ≥100 weekly PAI at all three HUNT waves had 3·9 kg (95% CI: 2·5 to 5·4) lower body weight gain at HUNT3 (Table 3).

Among both men and women obtaining ≥100 weekly PAI at both HUNT1 and HUNT3, there were no significant differences in body weight change between meeting or not meeting the physical activity recommendations (defined as 7·5 weekly MET-hours) (eTable 5 and eTable 6). Men with ≥100 PAI but not fulfilling the physical activity recommendations at HUNT1 and HUNT3 had similar body weight gain (−0·5 kg, 95% CI: −1·8 to 2·9) as men with ≥100 weekly PAI and meeting physical activity recommendations at both occasions (eTable 5). Similar data were observed for women (2·0 kg, 95% CI: −1·3 to 5·4) (eTable 6).

## 4 Discussion

In this large prospective study of relatively healthy participants spanned over more than two decades, we found an inverse association between PAI-score and weight gain in men and women. We observed an overall weight gain among participants over the years, however, those with high PAI-score had significantly less pronounced weight gain. Moreover, an increase in PAI-score from inactive to ≥100 weekly PAI after 11 and 22 years and maintaining a score of ≥100 PAI over 22 years were both associated with lower body weight gain compared with participants who remained inactive. Interestingly, these estimates appeared to be larger for women than for men. Indeed, women who either stayed active (≥100 weekly PAI) or became active over time had respectively 2·3 kg and 3·9 kg less body weight gain in the follow-up period, whereas the corresponding numbers for men were 1·2 kg and 1·6 kg. Of interest, previous studies have considered a 2·3 kg reduction in body weight as clinically significant [9,12,33,34]. Therefore, these data suggest that the PAI metric could be used as a suitable guidance in terms of physical activity levels to reduce excessive body weight gain over time.

A previous study using HUNT data showed that men and women meeting the recommended levels of physical activity had respectively 0·7 kg (95% CI: 0·5 to 0·9) and 0·5 kg (95% CI: 0·3 to 0·7) lower body weight gain over any 11-year period, compared with their inactive counterparts [9]. Our results suggest that men and women obtaining ≥100 weekly PAI at HUNT1 and HUNT3 had lower body weight gain regardless of following the physical activity guidelines. Interestingly, the findings of a recent publication suggest that ≥100 weekly PAI score may fit well with the upper limits of new physical activity recommendations for Americans,[6] and World Health Organization 2020 guidelines on physical activity and sedentary behaviour [7]. The physical activity recommendations define intensity in both absolute and relative terms with vaguely defined activity goals [6,16,17]. In comparison, the PAI is based on relative intensity and provides individuals with readily available feedback to track their activity levels using a single and straightforward activity metric. The algorithm also incorporates the fact that the higher the intensity of the activity, the shorter the time is needed to obtain 100 PAI. In practical terms, this is an important contribution to the physical activity science because earlier reports have shown that fewer activity sessions performed at higher intensities provide similar or larger health benefits compared with frequent, low intensity activity of longer durations [35,36]. Therefore, the PAI metric can be used as a guidance to secure appropriate weekly levels of physical activity and may contribute for weight gain prevention at population level.

Modern technologies in the healthcare sector have the potential to revolutionise health management both today and in the future. The PAI metric is integrated into wearable devices with a downloadable application (compatible with most Bluetooth enabled heart rate monitors) and is available worldwide. The PAI data may be shared between clinicians and patients/consumers and provides an opportunity for clinicians to track the activity levels of their patients and motivate them to increase their activity levels for most favourable health outcomes.

### 4.1 Strengths and limitations

The main strengths of the current study are the prospective design with 22 years of follow-up, the large sample size, and the extensive amount of data on possible confounding factors. Moreover, body weight was measured at all examinations using standardised methods [26]. Nevertheless, there are also some limitations. Due to the observational nature of the design, our analyses do not establish a causal relationship between PAI and body weight. Further, information used to estimate PAI, and data on confounding factors are all self-reported. These may, therefore, be subject to information bias. However, the questionnaires on physical activity in HUNT waves have been shown to provide acceptable reliability and validity [37,38]. The questions about physical activity were different in HUNT2 than those used in HUNT1 and HUNT3. However, in HUNT 3, both questionnaires that were used in HUNT1 and HUNT2 were available, and the cross-classification for PAI scores showed a fairly accurate placing of participants into the correct PAI category. In total, 63.5% and 66.3% of inactive men and women based on HUNT2 questions were correctly classified as being inactive by HUNT3 questions. While 76.2% and 79.8% of the men and women with PAI scores ≥100 estimated through HUNT2 questions were correctly classified into ≥100 PAI category by HUNT3 questions (data not shown). Moreover, previous studies using physical activity data from HUNT1 and HUNT2 have shown expected associations with morbidity and mortality [39,40]. Nonetheless, the comparison estimates between HUNT1 and HUNT3 are more robust and likely more unbiased than the comparisons with HUNT2, and a cautious interpretation is warranted. In the field of public health and exercise science, the use of activity-based wearables is projected to increase many folds in coming years together with the increasing resources to handle these data, which would make these kind of studies more feasible now than ever [41]. The PAI metric is relatively new compared with accelerometers or activity tracking wearables, therefore, future studies using objective measurements would certainly help to enhance our understanding of the role of PAI for promoting health and preventing diseases. Moreover, even though participants with CVD were excluded from our study sample, selection bias may still be present due to underlying diseases, and functional impairments among the study participants. The detailed information about diet was not available in this population. Although, mean energy intake was similar over an average of 5 years period in two separate studies in Norway, a large proportion of participants had higher intakes of saturated fats, and lower intakes of fibre and vitamin D than the nutritional recommendations [42,43]. However, it is suggested that despite the short-term benefits of weight loss related to dietary modifications, declined levels of physical activity may be the main reason for increased body weight over time rather than the changes in energy intake [44,45]. Furthermore, diet data from large scale population studies are hard to analyse and, therefore, should be interpreted with caution. Finally, participants included in this, and previous studies assessing PAI, are ethnically homogeneous [[20], [21], [22], [23],^25^]. Thus, one should be prudent before drawing definite inferences from the relationship between PAI and weight in other populations.

## 5 Conclusion

For both sexes, body weight increased over time across all PAI groups. The body weight gain was less pronounced in participants with high PAI-scores, and among those improving or maintaining a high PAI-score. The inverse association between weight gain and PAI was independent of adhering to physical activity recommendations. Finally, the PAI metric may aid in attenuating body weight gain, however, a multifactorial approach should be encouraged for successful body weight gain prevention.

Contributors*:* All authors have contributed with the conception of the work. Ulrik Wisløff, Javaid Nauman, Stian Lydersen, Ulf Ekelund, and Sophie Kieffer have contributed with analyses and interpretation of the data, and drafting of the work, while all authors have revised the work and contributed with intellectual content. Sigurd Steinshamn has monitored adherence to the design and statistical analyses. Kieffer, Nauman, and Lydersen had full access to all the data in the study and takes responsibility for the integrity of the data and the accuracy of the data analyses. Acquisition of data: Kieffer, Nauman, Syverud, Selboskar. Analysis and interpretation of data: Kieffer, Lydersen, Nauman, Ekelund, Wisløff. Drafting of the manuscript: Kieffer, Syverud, Selboskar, Lydersen, Nauman, Ekelund, Wisløff. Critical revision of the manuscript for intellectual content: Kieffer, Syverud, Selboskar, Lydersen, Nauman, Ekelund, Wisløff. Study supervision: Wisløff, Nauman.

Declaration of interest: Professor Ulrik Wisløff is the inventor of PAI and serves as a scientific consultant for PAI Health Inc, a software company that develops applications using data from heart rate monitors to display PAI. Due to this potential conflicting interest, we are grateful to Professor Sigurd Steinshamn, at the Department of Circulation and Medical Imaging, Faculty of Medicine and Health Sciences, NTNU for monitoring adherence to the design, and statistical analyses in the current study. There are no further disclosures or conflict of interest to report.

Acknowledgements: The Trøndelag Health Study (HUNT) is a collaboration between HUNT Research Centre (Faculty of Medicine and Health Sciences and the Norwegian University of Science and Technology, NTNU), Trøndelag County Council, Central Norway Regional Health Authority, and the Norwegian Institute of Public Health. The HUNT study management has provided data used in the analyses. We are greatly appreciative of the participants in the HUNT study, and the management of the HUNT study for providing these data.

Funding: The study was funded by grants from the Norwegian Research Council and the Liaison Committee between the Central Norway Regional Health Authority and the Norwegian University of Science and technology. The funding organizations had no role in the design and execution of the study, in the collection, analyses or interpretation of the data. Further, they had no role in the preparation, review or approval of the manuscript.

Data sharing statement: Researchers associated with Norwegian research institutes can apply for the use of HUNT material: data and samples - given approval by a Regional Committee for Medical and Health Research Ethics. Researchers from other countries are welcome to apply in cooperation with a Norwegian Principle Investigator. Access to the requested HUNT material is given after the application is approved of by HUNT Data Access Committee and an agreement is signed. The agreement gives the researcher(s) the right to research a specific topic for a limited time period and to publish a decided upon number of articles.

## Appendix Supplementary materials

Image, application 1

Image, application 2
