# Supplementary material for: Association between Personal Activity Intelligence (PAI) and body weight in a population free from cardiovascular disease – The HUNT study
Source: Lancet Reg Health Eur. 2021 Mar 21;5:100091. doi: 10.1016/j.lanepe.2021.100091 (PMC8454800; doi:10.1016/j.lanepe.2021.100091)
Supplement: Supplementary file 1 [file mmc1.docx]

**eTable1.** Characteristics of study participants according to participation in HUNT waves.

**eTable 2.** Expected weight (CI) in kg, by PAI categories at HUNT1, HUNT2 and HUNT3.

**eTable 3**. Difference in body weight (95% CI) in kilograms associated with Personal Activity intelligence.

**eTable 4.** Complete case analysis: difference in body weight (95% CI) in kilogram and interaction estimates between body weight and time by PAI categories.

**eTable 5.** Changes in body weight in kilogram between HUNT1 and HUNT3 among men according to PAI and 7.5 METs-hours.

**eTable 6.** Changes in body weight in kilogram between HUNT1 and HUNT3 among women according to PAI and 7.5 METs-hours.

**eFigure 1.** Flow of participants in the complete case analyses.
