# Supplementary material for: Association between Personal Activity Intelligence (PAI) and body weight in a population free from cardiovascular disease – The HUNT study
Source: Lancet Reg Health Eur. 2021 Mar 21;5:100091. doi: 10.1016/j.lanepe.2021.100091 (PMC8454800; doi:10.1016/j.lanepe.2021.100091)
Supplement: Supplementary file 2 [file mmc2.docx]

Online Supplemental Material

**Page 2: eTable1.** Characteristics of study participants according to participation in HUNT waves.

**Page 3: eTable 2.** Expected weight (CI) in kg, by PAI categories at HUNT1, HUNT2 and HUNT3.

**Page 4: eTable 3**. Difference in body weight (95% CI) in kilograms associated with Personal Activity intelligence.

**Page 5: eTable 4.** Complete case analysis: difference in body weight (95% CI) in kilogram and interaction estimates between body weight and time by PAI categories.

**Page 6: eTable 5.** Changes in body weight in kilogram between HUNT1 and HUNT3 among men according to PAI and 7.5 METs-hours.

**Page 7: eTable 6.** Changes in body weight in kilogram between HUNT1 and HUNT3 among women according to PAI and 7.5 METs-hours.

**Page 8**: **eFigure 1.** Flow of participants in the complete case analyses.

**eTable 1. Characteristics of study participants according to participation in HUNT waves.**

|  | HUNT1^a^ | | |  | HUNT2^b^ | | |  | HUNT3^c^ | | |
| --- | --- | --- | --- | --- | --- | --- | --- | --- | --- | --- | --- |
|  | HUNT1 | HUNT1 & HUNT2 | HUNT1 & HUNT3 |  | HUNT2 | HUNT1 & HUNT2 | HUNT2 & HUNT3 |  | HUNT3 | HUNT1 & HUNT3 | HUNT2 & HUNT3 |
|  | (N= 65 892) | (N= 40 627) | (N=26 837) |  | (N= 57 979) | (N= 40 627) | (N= 33 451) |  | (N=46 636) | (N= 26 837) | (N=33 451) |
| PAI, N (%) |  |  |  |  |  |  |  |  |  |  |  |
| Inactive | 21 482 (32.6) | 13 394 (33.0) | 8 846 (33.0) |  | 3 954 (6.8) | 2 924 (7.2) | 1 769 (5.3) |  | 9 898 (21.2) | 5 490 (20.5) | 6 830 (20.4) |
| ≤50 | 15 526 (23.6) | 9 863 (24.3) | 5 859 (21.8) |  | 22 562 (38.9) | 17 306 (42.6) | 13 319 (39.8) |  | 12 175 (26.1) | 8 224 (30.6) | 9 504 (28.4) |
| 51-99 | 4 848 (7.4) | 3 431 (8.5) | 2 461 (9.2) |  | 8 905 (15.4) | 5 699 (14.0) | 5 797 (17.3) |  | 5 166 (11.1) | 2 926 (10.9) | 3 743 (11.2) |
| 100-199 | 4 197 (6.4) | 3 042 (7.5) | 2 312 (8.6) |  | 9 127 (15.7) | 5 561 (13.7) | 6 104 (18.3) |  | 5 014 (10.8) | 2 752 (10.3) | 3 522 (10.5) |
| ≥200 | 4 951 (7.5) | 3 463 (8.5) | 2 701 (10.1) |  | 7 646 (13.2) | 3 979 (9.8) | 4 691 (14.0) |  | 12 901 (27.7) | 6 395 (23.8) | 8 700 (26.0) |
| Female, N (%) | 34 616 (52.5) | 22 477 (55.3) | 14 951 (55.7) |  | 31 723 (54.7) | 22 477 (55.3) | 19 030 (56.9) |  | 26 137 (56.0) | 14951 (55.7) | 19 030 (56.9) |
| Age years, mean (SD) | 47.5 (17.7) | 43.4 (14.1) | 38.5 (10.6) |  | 48.2 (16.8) | 54.9 (14.1) | 45.6 (13.0) |  | 51.7 (15.6) | 61.1 (10.6) | 56.8 (13.0) |
| Weight kg, mean (SD) | 72.0 (12.9) | 71.7 (12.5) | 71.0 (12.2) |  | 76.2 (14.0) | 76.6 (13.8) | 76.2 (13.6) |  | 79.1 (15.2) | 79.3 (14.6) | 79.3 (14.9) |
| Height cm, mean (SD) | 169.6 (9.4) | 170.0 (9.1) | 170.9 (8.9) |  | 170.3 (9.4) | 169.5 (9.3) | 170.8 (9.0) |  | 170.7 (9.2) | 169.6 (9.2) | 170.1 (9.2) |
| BMI, kg/m^2^, N (%) |  |  |  |  |  |  |  |  |  |  |  |
| < 18.5 | 908 (1.4) | 472 (1.2) | 325 (1.2) |  | 418 (0.7) | 225 (0.6) | 190 (0.6) |  | 286 (0.6) | 115 (0.4) | 146 (0.4) |
| 18.5 - 24.9 | 34 422 (52.2) | 22 839 (56.2) | 16 659 (62.1) |  | 23 229 (40.1) | 14 446 (35.6) | 13 916 (41.6) |  | 15 271 (32.8) | 7 469 (27.8) | 9 949 (29.7) |
| 25.0 - 29.9 | 21 750 (33.0) | 13 286 (32.7) | 7 899 (29.4) |  | 24 319 (41.9) | 18 190 (44.8) | 14 527 (43.4) |  | 20 387 (43.7) | 12577 (46.9) | 15 377 (46.0) |
| ≥ 30.0 | 6 472 (9.8) | 3 432 (8.5) | 1 502 (5.6) |  | 9 097 (15.7) | 7 078 (17.4) | 4 655 (13.9) |  | 10 384 (22.3) | 6 519 (24.3) | 7 798 (23.3) |
| Alcohol consumption/2 weeks, N (%) | | |  |  |  |  |  |  |  |  |  |
| Abstainer | 5 998 (9.1) | 2 944 (7.3) | 1 454 (5.4) |  | 6 387 (11.0) | 5 233 (12.9) | 2 718 (8.1) |  | 1 741 (3.7) | 1 205 (4.5) | 1 363 (4.1) |
| <1 time | 23 377 (35.5) | 14 862 (36.6) | 9 208 (34.3) |  | 18 923 (32.6) | 13 829 (34.0) | 11 055 (33.1) |  | 16 425 (35.2) | 9 244 (34.4) | 11 544 (34.5) |
| 1-4 times | 19 962 (30.3) | 14 203 (35.0) | 10 664 (39.7) |  | 25 832 (44.6) | 16 144 (39.7) | 16 255 (48.6) |  | 20 364 (43.7) | 11290 (42.1) | 14 423 (43.1) |
| ≥ 5 times | 3 045 (4.6) | 1 899 (4.7) | 1 183 (4.4) |  | 1 681 (2.9) | 1 259 (3.1) | 1 018 (3.0) |  | 6 892 (14.8) | 4 234 (15.8) | 5 174 (15.5) |
| Smoking status, N (%) | |  |  |  |  |  |  |  |  |  |  |
| Never | 21 926 (33.3) | 15 001 (36.9) | 10 071 (37.5) |  | 25 224 (43.5) | 16 254 (40.0) | 15 455 (46.2) |  | 19 876 (42.6) | 10186 (38.0) | 13 892 (41.5) |
| Former | 11 645 (17.7) | 8 073 (19.9) | 5 454 (20.3) |  | 14 485 (25.0) | 11 586 (28.5) | 8 999 (26.9) |  | 17 559 (37.7) | 11050 (41.2) | 13 015 (38.9) |
| Current | 17 857 (27.1) | 11 198 (27.6) | 7 130 (26.6) |  | 16 702 (28.8) | 11 642 (28.7) | 8 680 (26.0) |  | 7 945 (17.0) | 4 697 (17.5) | 5 543 (16.6) |
| Hypertension status, N (%) | |  |  |  |  |  |  |  |  |  |  |
| yes | 29 709 (45.1) | 15 404 (37.9) | 7 620 (28.4) |  | 23 643 (40.8) | 19 960 (49.1) | 11 663 (34.9) |  | 17 100 (36.7) | 13654 (50.9) | 14 557 (43.5) |
| No | 34 331 (52.1) | 24 640 (60.7) | 18 762 (69.9) |  | 33 659 (58.1) | 20 178 (49.7) | 21 674 (64.8) |  | 29 250 (62.7) | 13065 (48.7) | 18 736 (56.0) |
| Diabetes status, N (%) | |  |  |  |  |  |  |  |  |  |  |
| yes | 1 455 (2.2) | 344 (0.9) | 108 (0.4) |  | 1 360 (2.4)) | 1 196 (2.9) | 420 (1.3) |  | 1 730 (3.7) | 1 392 (5.2) | 1 451 (4.3) |
| No | 64 270 (97.5) | 40 235 (99.0) | 26 699 (99.5) |  | 56 200 (96.9) | 39 037 (96.1) | 32 982 (98.6) |  | 44 882 (96.2) | 25430 (94.8) | 31 982 (95.6) |
| Occupational PA, N (%) | |  |  |  |  |  |  |  |  |  |  |
| Sedentary | 5 293 (8.0) | 3 832 (9.4) | 2 832 (10.6) |  | 13 617 (23.5) | 9 131 (22.5) | 8 442 (25.2) |  | 11 844 (25.4) | 5 880 (21.9) | 8 039 (24.0) |
| Walk and lift | 29 035 (44.1) | 22 075 (54.3) | 15 982 (59.6) |  | 26 635 (45.9) | 17 698 (43.6) | 17 602 (52.6) |  | 22 718 (48.7) | 11680 (43.5) | 15 521 (46.4) |
| Heavy | 3 604 (5.5) | 2 582 (6.4) | 1 799 (6.7) |  | 5 866 (10.1) | 4 111 (10.1) | 3 701 (11.1) |  | 3 830 (8.2) | 2 308 (8.6) | 2 829 (8.5) |
|  |  |  |  |  |  |  |  |  |  |  |  |
| PAI: Personal Activity Intelligence, N: number, SD: standard deviation, BMI: Body Mass Index, cm: centimeter, kg: kilograms, m: meters.  ^a^Data is reported for HUNT1. ^b^Data is reported for HUNT2. ^c^Data is reported for HUNT3. | | | | | | | | | | | |

| **eTable 2. Expected weight (CI) in kg, by PAI categories at HUNT1, HUNT2 and HUNT3** | | | | | |  |  |  |  |  |
| --- | --- | --- | --- | --- | --- | --- | --- | --- | --- | --- |
|  |  | Men | |  |  |  | Women | |  |  |
|  | PAI | N | HUNT1 | HUNT2 | HUNT3 |  | N | HUNT1 | HUNT2 | HUNT3 |
| **Model 1** | Inactive |  | 78.2 (78.0 to 78.4) | 83.2 (82.9 to 83.5) | 88.2 (88.0 to 88.4) |  |  | 66.2 (66.0 to 66.4) | 70.4 (70.0 to 70.7) | 73.8 (73.5 to 74.0) |
|  | ≤50 |  | 78.7 (78.5 to 79.0) | 83.1 (83.0 to 83.3) | 86.4 (86.1 to 86.6) |  |  | 66.6 (66.4 to 66.8) | 70.7 (70.6 to 70.9) | 72.7 (72.5 to 72.9) |
|  | 51-99 |  | 78.3 (78.0 to 78.7) | 83.5 (83.3 to 83.7) | 87.7 (87.4 to 88.0) |  |  | 65.6 (65.3 to 65.9) | 71.1 (70.8 to 71.4) | 73.0 (72.7 to 73.3) |
|  | 100-199 |  | 78.3 (78.0 to 78.5) | 83.3 (83.1 to 83.5) | 87.2 (87.0 to 87.5) |  |  | 65.4 (64.9 to 65.9) | 70.4 (70.2 to 70.6) | 74.0 (73.5 to 74.5) |
|  | ≥200 |  | 77.1 (76.8 to 77.4) | 82.1 (81.9 to 82.4) | 85.5 (85.2 to 85.7) |  |  | 65.9 (65.6 to 66.2) | 69.9 (69.6 to 70.1) | 72.8 (72.6 to 73.0) |
|  |  | 40 037 |  |  |  |  | 45 206 |  |  |  |
| **Model 2** | Inactive |  | 78.6 (78.4 to 78.8) | 83.5 (83.1 to 83.8) | 88.1 (87.9 to 88.3) |  |  | 66.3 (66.1 to 66.5) | 70.4 (70.0 to 70.7) | 73.9 (73.7 to 74.2) |
|  | ≤50 |  | 79.0 (78.8 to 79.3) | 83.4 (83.2 to 83.6) | 86.1 (85.9 to 86.4) |  |  | 66.6 (66.4 to 66.8) | 70.7 (70.6 to 70.9) | 72.6 (72.4 to 72.8) |
|  | 51-99 |  | 78.6 (78.3 to 79.0) | 83.7 (83.5 to 83.9) | 87.5 (87.1 to 87.8) |  |  | 65.5 (65.2 to 65.8) | 71.1 (70.8 to 71.4) | 72.9 (72.6 to 73.2) |
|  | 100-199 |  | 78.4 (78.1 to 78.7) | 83.4 (83.2 to 83.6) | 86.9 (86.7to- 87.2) |  |  | 65.3 (64.9 to 65.8) | 70.4 (70.1 to 70.6) | 73.8 (73.2 to 74.3) |
|  | ≥200 |  | 77.3 (77.0 to 77.6) | 82.2 (81.9 to 82.5) | 85.1 (84.9 to 85.4) |  |  | 65.8 (65.4 to 66.1) | 69.8 (69.6 to 70.1) | 72.5 (72.3 to 72.7) |
|  |  | 38 557 |  |  |  |  | 42 800 |  |  |  |
| **Model 3** | Inactive |  | 79.4 (79.2 to 79.6) | 84.2 (83.8 to 84.6) | 88.5 (88.2 to 88.7) |  |  | 66.1 (65.9 to 66.3) | 70.8 (70.4 to 71.3) | 74.3 (74.1 to 74.6) |
|  | ≤50 |  | 79.6 (79.3 to 79.9) | 84.0 (83.8 to 84.2) | 86.8 (86.6 to 87.1) |  |  | 65.9 (65.7 to 66.1) | 70.7 (70.5 to 70.8) | 73.1 (72.8 to 73.3) |
|  | 51-99 |  | 79.5 (79.2 to 79.9) | 84.1 (83.9 to 84.4) | 87.8 (87.4 to 88.1) |  |  | 65.3 (65.0 to 65.7) | 70.9 (70.6 to 71.2) | 73.3 (73.0 to 73.6) |
|  | 100-199 |  | 79.1 (78.9 to 79.4) | 83.8 (83.6 to 84.0) | 87.0 (86.7 to 87.3) |  |  | 65.3 (64.8 to 65.8) | 70.2 (70.0 to 70.5) | 73.4 (72.8 to 73.4) |
|  | ≥200 |  | 78.1 (77.8 to 78.4) | 82.7 (82.5 to 83.0) | 85.4 (85.1 to 85.7) |  |  | 66.0 (65.6 to 66.3) | 69.7 (69.5 to 70.0) | 72.1 (71.9 to 72.3) |
|  |  | 32 764 |  |  |  |  | 35 746 |  |  |  |

PAI: Personal Activity Intelligence, HUNT: The Trøndelag Health Study, CI: confidence interval, kg: kilogram, N: number

Model 1 adjusted for age

Model 2 further adjusted for diabetes status (yes or no), smoking status (never, former, current), alcohol consumption (abstainers, <1 time/2 weeks, 1-4 times/2weeks or ≥5 times/2weeks).

Model 3 further adjusted for occupational physical activity (sedentary, walk and lift and heavy).

**eTable 3. Difference in body weight (95% CI) in kilograms associated with Personal Activity intelligence.**

|  |  | Men |  |  |  |  | Women |  |  |  |
| --- | --- | --- | --- | --- | --- | --- | --- | --- | --- | --- |
|  | PAI | N | HUNT1 | HUNT2 | HUNT3 |  | N | HUNT1 | HUNT2 | HUNT3 |
| **Model 1** | 0 |  | Ref | 5.0 (4.6 to 5.3) | 10.0 (9.7 to 10.2) |  |  | Ref | 4.2 (3.8 to 4.5) | 7.6 (7.3 to 7.9) |
|  | 1-50 |  | Ref | 4.4 (4.2 to 4.7) | 7.6 (7.3 to 7.9) |  |  | Ref | 4.1 (3.9 to 4.3) | 6.1 (5.8 to 6.3) |
|  | 51-99 |  | Ref | 5.2 (4.8 to 5.6) | 9.4 (8.9 to 9.8) |  |  | Ref | 5.5 (5.2 to 5.9) | 7.4 (7.0 to 7.8) |
|  | 100-199 |  | Ref | 5.1 (4.8 to 5.4) | 9.0 (8.6 to 9.3) |  |  | Ref | 5.0 (4.5 to 5.5) | 8.6 (7.9 to 9.3) |
|  | ≥200 |  | Ref | 5.0 (4.7 to 5.4) | 8.3 (8.0 to 8.7) |  |  | Ref | 4.0 (3.6 to 4.4) | 6.9 (6.5 to 7.2) |
|  |  | 40 037 |  |  |  |  | 45 206 |  |  |  |
| **Model 2** | 0 |  | Ref | 4.9 (4.5 to 5.2) | 9.5 (9.2 to 9.7) |  |  | Ref | 4.1 (3.7 to 4.4) | 7.6 (7.3 to 7.9) |
|  | 1-50 |  | Ref | 4.4 (4.1 to 4.6) | 7.1 (6.8 to 7.4) |  |  | Ref | 4.2 (4.0 to 4.4) | 6.1 (5.8 to 6.3) |
|  | 51-99 |  | Ref | 5.1 (4.7 to 5.4) | 8.8 (8.4 to 9.3) |  |  | Ref | 5.6 (5.2 to 6.0) | 7.4 (7.0 to 7.8) |
|  | 100-199 |  | Ref | 5.0 (4.7 to 5.3) | 8.5 (8.2 to 8.9) |  |  | Ref | 5.0 (4.5 to 5.5) | 8.4 (7.7 to 9.1) |
|  | ≥200 |  | Ref | 4.9 (4.6 to 5.3) | 7.9 (7.5 to 8.2) |  |  | Ref | 4.1 (3.7 to 4.4) | 6.7 (6.4 to 7.1) |
|  |  | 38 557 |  |  |  |  | 42 800 |  |  |  |
| **Model 3** | 0 |  | Ref | 4.8 (4.4 to 5.2) | 9.1 (8.8 to 9.4) |  |  | Ref | 4.7 (4.2 to 5.2) | 8.2 (7.9 to 8.6) |
|  | 1-50 |  | Ref | 4.4 (4.1 to 4.7) | 7.2 (6.9 to 7.6) |  |  | Ref | 4.8 (4.5 to 5.0) | 7.2 (6.9 to 7.5) |
|  | 51-99 |  | Ref | 4.6 (4.2 to 5.0) | 8.2 (7.8 to 8.7) |  |  | Ref | 5.6 (5.2 to 6.0) | 8.0 (7.5 to 8.4) |
|  | 100-199 |  | Ref | 4.7 (4.3 to 5.0) | 7.9 (7.5 to 8.2) |  |  | Ref | 4.9 (4.4 to 5.5) | 8.1 (7.3 to 8.8) |
|  | ≥200 |  | Ref | 4.6 (4.3 to 5.0) | 7.3 (6.9 to 7.7) |  |  | Ref | 3.8 (3.4 to 4.2) | 6.2 (5.8 to 6.6) |
|  |  | 32 764 |  |  |  |  | 35 746 |  |  |  |

PAI: Personal Activity Intelligence, HUNT: The Trøndelag Health Study, CI: confidence interval, N: number

Model 1 adjusted for age.

Model 2 was further adjusted for diabetes status (yes or no), smoking status (never, former, current) and alcohol consumption (abstainers, <1 time/2 weeks, 1-4 times/2weeks or ≥5 times/2weeks).

Model 3 was further adjusted for occupational physical activity (sedentary, walk and lift and heavy).

**eTable 4. Complete case analysis: difference in body weight (95% CI) in kilogram and interaction estimates between body weight and time by PAI categories.**

|  |  | Men |  |  |  | Women |  |  |
| --- | --- | --- | --- | --- | --- | --- | --- | --- |
|  | PAI | HUNT1 | HUNT2 | HUNT3 |  | HUNT1 | HUNT2 | HUNT3 |
| **Model 1** | Inactive | Ref | 6.8 (6.0 to 7.5) | 10.3 (9.4 to 11.1) |  | Ref | 6.3 (5.4 to 7.2) | 9.0 (8.1 to 10.0) |
|  | ≤50 | 0.5 (0.1 to 0.9) | *-0.8 (-1.5 to 0)* | *-1.5 (-2.1 to -1.0)* |  | 0.1 (-0.3 to 0.4) | *-0.8 (-1.6 to 0.1)* | *-1.7 (-2.2 to -1.1)* |
|  | 51-99 | 0.4 (-0.1 to 0.9) | *-0.9 (-1.7 to -0.1)* | *-0.7 (-1.4 to 0)* |  | -0.1 (-0.6 to 0.3) | *-0.4 (-1.4 to 0.5)* | *-1.4 (-2.5 to -0.4)* |
|  | 100-199 | 0.4 (0 to 0.8) | *-1.3 (-2.1 to -0.6)* | *-1.5 (-2.0 to -1.0)* |  | 0.1 (-0.6 to 0.8) | *-1.4 (-2.5 to -0.4)* | *-1.3 (-2.4 to -0.3)* |
|  | ≥200 | -0.6 (-1.0 to -0.1) | *-1.0 (-1.8 to -0.2)* | *-1.7 (-2.3 to -1.2)* |  | 0.1 (0.5 to 1.5) | *-2.7 (-3.6 to -1.7* | *-3.3 (-3.9 to -2.6)* |
|  |  |  |  |  |  |  |  |  |
| **Model 2** | Inactive | Ref | 6.7 (6.0 to 7.4) | 10.0 (9.2 to 10.8) |  | Ref | 6.4 (5.5 to 7.3) | 9.1 (8.2 to 10.0) |
|  | ≤50 | 0.5 (0.1 to 0.9) | *-0.8 (-1.5 to -0.1)* | *-1.6 (-2.1 to -1.1)* |  | -0.1 (-0.4 to 0.3) | *-0.7 (-1.6 to 0.2)* | *-1.6 (-2.2 to -1.0)* |
|  | 51-99 | 0.3 (-0.2 to 0.7) | *-0.8 (-1.5 to 0)* | *-0.6 (-1.3 to 0.1)* |  | -0.3 (-0.8 to 0.2) | *-0.4 (-1.4 to 0.6)* | *-1.4 (-2.1 to -0.6)* |
|  | 100-199 | 0.2 (-0.2 to 0.6) | *-1.2 (-1.9 to -0.4)* | *-1.4 (-1.9 to -0.9)* |  | -0.1 (-0.8 to 0.6) | *-1.5 (-2.5 to -0.4)* | *-1.3 (-2.3 to -0.2)* |
|  | ≥200 | -0.8 (-1.3 to -0.4) | *-0.9 (-1.7 to -0.1)* | *-1.7 (-2.2 to -1.1)* |  | 0.7 (0.2 to 1.2) | *-2.6 ((-3.5 to -1.6)* | *-3.2 (-3.8 to -2.5)* |
|  |  |  |  |  |  |  |  |  |
| **Model 3** | Inactive | Ref | 6.7 (6.0 to 7.4) | 9.9 (9.1 to 10.7)*^a^* |  | Ref | *6.3 (5.5 to 7.2)* | *9.0 (8.1 to 10.0)* |
|  | ≤50 | 0.5 (0.1 to 0.9) | *-0.9 (-1.6 to -0.2)* | *-1.7 (-2.2 to -1.1)* |  | -0.1 (-0.4 to 0.3) | *-0.7 (-1.6 to 0.1)* | *-1.6 (-2.2 to -1.0)* |
|  | 51-99 | 0.2 (-0.2 to 0.7) | *-0.8 (-1.6 to -0.1)* | *-0.6 (-1.3 to 0)* |  | -0.3 (-0.7 to 0.2) | *-0.4 (-1.4 to 0.5)* | *-1.4 (-2.1 to -0.7)* |
|  | 100-199 | 0.1 (-0.3 to 0.5) | *-1.2 (-2.0 to -0.5)* | *-1.4 (-1.9 to -0.8)* |  | -0.1 (-0.8 to 0.6) | *-1.5 (-2.5 to -0.4)* | *-1.3 (-2.3 to -0.2)* |
|  | ≥200 | -0.9 (-1.3 to -0.5)*^a^* | *-0.9 (-1.7 to 0.1)* | *-1.6 (-2.2 to -1.0) ^a^* |  | 0.7 (0.2 to 1-2) | *-2.6 (-3.5 to -1.6)* | *-3.2 (-3.8 to -2.5)* |

PAI: Personal Activity Intelligence, HUNT: The Trøndelag Health Study, CI: confidence interval, kg: kilogram

Model 1 adjusted for age

Model 2 further adjusted for diabetes status (yes or no), smoking status (never, former, current), alcohol consumption (abstainers, <1 time/2 weeks, 1-4 times/2weeks or ≥5 times/2weeks.

Model 3 further adjusted for occupational physical activity (sedentary, walk and lift and heavy).

Numbers in italic are interactions estimates between PAI category and HUNT wave. The interpretation is as follows: ^a^Compared with the inactive group at HUNT1, those obtaining ≥200 at HUNT1 has 0.9 kg lower body weight at HUNT1. Compared to the same reference group, inactive men had 9.9 kg higher body weight at HUNT3, and men obtaining ≥200 at HUNT3 had 1.6 kg lower body weight increase at HUNT3 (interaction estimate).

Finally, the expected body weight in the ≥200 PAI group can then be calculated with the following equation: body weight in reference group – 0.9 + 9.9 – 1.6.

| PAI | | n | weight change (CI) |
| --- | --- | --- | --- |
| HUNT1 | HUNT3 |  |  |
| **Men** |  |  |  |
| ≥100 PAI & ≥7.5 MET-h | ≥100 PAI & ≥7.5 MET-h | 1 205 | Ref |
| ≥100 PAI & ≥7.5 MET-h | ≥100 PAI & <7.5 MET-h | 109 | 1.0 (-0.5 to 2.4) |
| ≥100 PAI & ≥7.5 MET-h | <100 PAI & ≥7.5 MET-h | 112 | 1.2 (-0.3 to 2.6) |
| ≥100 PAI & ≥7.5 MET-h | <100 PAI & <7.5 MET-h | 579 | 2.2 (1.5 to 3.0) |
| ≥100 PAI & <7.5 MET-h | ≥100 PAI & ≥7.5 MET-h | 240 | 0.6 (-0.5 to 1.6) |
| ≥100 PAI & <7.5 MET-h | ≥100 PAI & <7.5 MET-h | 40 | -0.5 (-2.9 to 1.8) |
| ≥100 PAI & <7.5 MET-h | <100 PAI & ≥7.5 MET-h | 37 | -0.3 (-2.7 to 2.2) |
| ≥100 PAI & <7.5 MET-h | <100 PAI & <7.5 MET-h | 253 | 2.0 (1.0 to 3.0) |
| <100 PAI & ≥7.5 MET-h | ≥100 PAI & ≥7.5 MET-h | 42 | 0.7 (-1.6 to 3.0) |
| <100 PAI & ≥7.5 MET-h | ≥100 PAI & <7.5 MET-h | 1 | 3.3 (-11.4 to 17.9) |
| <100 PAI & ≥7.5 MET-h | <100 PAI & ≥7.5 MET-h | 23 | 1.8 (-1.3 to 4.9) |
| <100 PAI & ≥7.5 MET-h | <100 PAI & <7.5 MET-h | 57 | 1.1 (-0.8 to 3.1) |
| <100 PAI & <7.5 MET-h | ≥100 PAI & ≥7.5 MET-h | 1 300 | 0.6 (0 to 1.2) |
| <100 PAI & <7.5 MET-h | ≥100 PAI & <7.5 MET-h | 281 | 1.0 (0.1 to 2.0) |
| <100 PAI & <7.5 MET-h | <100 PAI & ≥7.5 MET-h | 375 | 0.1 (-0.8 to 1.0) |
| <100 PAI & <7.5 MET-h | <100 PAI & <7.5 MET-h | 3 698 | 1.6 (1.2 to 2.1) |

**eTable 5. Changes in body weight in kilogram between HUNT1 and HUNT3 among men according to PAI and 7.5 METs-hours**

PAI; Personal Activity Intelligence, MET; metabolic Equivalent of Tasks, HUNT; The Trøndelag Health Study, N; number, CI; confidence interval

Adjusted for age.

| PAI | | N | weight change (CI) |
| --- | --- | --- | --- |
| HUNT1 | HUNT3 |  |  |
| **Women** |  |  |  |
| ≥100 PAI & ≥7.5 MET-h | ≥100 PAI & ≥7.5 MET-h | 739 | Ref |
| ≥100 PAI & ≥7.5 MET-h | ≥100 PAI & <7.5 MET-h | 40 | 2.0 (-0.7 to 4.6) |
| ≥100 PAI & ≥7.5 MET-h | <100 PAI & ≥7.5 MET-h | 120 | 0.2 (-1.5 to 1.8) |
| ≥100 PAI & ≥7.5 MET-h | <100 PAI & <7.5 MET-h | 356 | 2.6 (1.6 to 3.7) |
| ≥100 PAI & <7.5 MET-h | ≥100 PAI & ≥7.5 MET-h | 247 | 0.5 (-0.7 to 1.7) |
| ≥100 PAI & <7.5 MET-h | ≥100 PAI & <7.5 MET-h | 25 | 2.0 (-1.3 to 5.4) |
| ≥100 PAI & <7.5 MET-h | <100 PAI & ≥7.5 MET-h | 51 | 0 (-2.3 to 2.4) |
| ≥100 PAI & <7.5 MET-h | <100 PAI & <7.5 MET-h | 204 | 3.2 (1.9 to 4.5) |
| <100 PAI & ≥7.5 MET-h | ≥100 PAI & ≥7.5 MET-h | 79 | -0.3 (-2.3 to 1.6) |
| <100 PAI & ≥7.5 MET-h | ≥100 PAI & <7.5 MET-h | 4 | 3.9 (-4.3 to 12.2) |
| <100 PAI & ≥7.5 MET-h | <100 PAI & ≥7.5 MET-h | 87 | 2.6 (0.7 to 4.5) |
| <100 PAI & ≥7.5 MET-h | <100 PAI & <7.5 MET-h | 137 | 2.2 (0.6 to 3.7) |
| <100 PAI & <7.5 MET-h | ≥100 PAI & ≥7.5 MET-h | 2 273 | 1.5 (0.8 to 2.2) |
| <100 PAI & <7.5 MET-h | ≥100 PAI & <7.5 MET-h | 260 | 1.9 (0.7 to 3.0) |
| <100 PAI & <7.5 MET-h | <100 PAI & ≥7.5 MET-h | 799 | 0.9 (0 to 1.7) |
| <100 PAI & <7.5 MET-h | <100 PAI & <7.5 MET-h | 4 858 | 2.8 (2.1 to 3.4) |

**eTable 6. Changes in body weight in kilogram between HUNT1 and HUNT3 among women according to PAI and 7.5 METs-hours.**

PAI; Personal Activity Intelligence, MET; metabolic Equivalent of Tasks, HUNT; The Trøndelag Health Study, N; number, CI; confidence interval

Adjusted for age.

**eFigure 1. Flow of participants in the complete case analyses.**


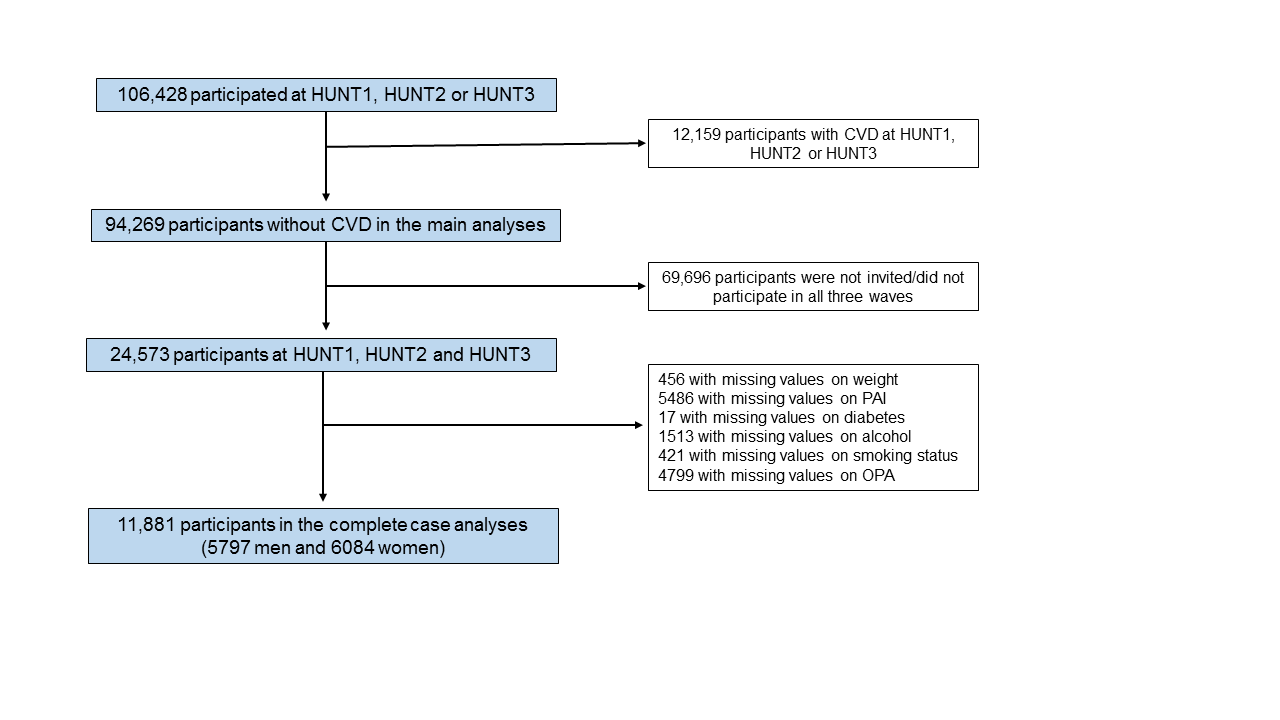


PAI: Personal Activity Intelligence,

HUNT: The Trøndelag Health Study,

CVD: cardiovascular disease

OPA: occupational physical activity
